# 

**Published:** 1872-07

**Authors:** 


### CONTENTS:

ORIGINAL COMMUNICATIONS:

ARTICLE I. Syphilitic Lesions of the Viscera ........ 385

ART. II. Case of Hemorrhagic Variola ........ 401

ART. III. Case of Malignant Epulis of the Lower Jaw—with Operation by Prof. Freer ........ 402

PROCEEDINGS OF SOCIETIES:

Annual Meeting of Military Tract Med. Association, at Galesburg, Ill. 407

SELECTIONS:

Results of Experiments on Pyæmia.. 409
Small-Pox—Vaccination and Revaccination—False Ideas ........ 418
On the Treatment of Asthma ........ 424
Diseases of the Muscular Walls of the Heart ........ 425
Strangulated Hernia ........ 434
Faber's Talking Machine ........ 437

EDITORS' BOOK TABLE ........ 441

ITEMS, NEWS AND GOSSIP ........ 443

LOOT ........ 445

